# Inflammatory bowel disease: invisible and unpredictable

**DOI:** 10.1016/j.ebiom.2024.105499

**Published:** 2024-12-11

**Authors:** eBioMedicine

Inflammatory bowel disease (IBD) is a term that includes Crohn's disease and ulcerative colitis, two related but distinct diseases. An invisible, yet life-altering, illness is how many people describe their lived experience; “if you don't look sick, you can't be that sick” is what they often hear. People with IBD often report that their gastrointestinal symptoms are uncomfortable or embarrassing to talk about with anyone other than their closest friends and family and the alternating periods of remission and relapse resemble a roller coaster, with the unpredictability of IBD affecting quality of life. Many people wait years for initial diagnosis or to gain access to newer, more effective treatments, an issue that, in some countries, is exacerbated by insurance coverage and policies. These concerns are highlighted at the annual Crohn's and Colitis Awareness Week during Dec 1–7 each year, and emphasise the urgent need for research on the causes, pathophysiology, diagnosis, and treatment of IBD.

Although genetic factors have long been implicated in the aetiology of IBD and genome-wide association studies (GWAS) have identified numerous associated loci, delineating the underlying biology has so far been elusive. In June, 2024, a study published in *Nature* by Stankey and colleagues was hailed as a breakthrough in the field. The authors investigated a gene-desert region in chromosome 21 that had previously been linked to IBD and identified the transcription factor *ETS2* as the causal gene involved. *ETS2* regulates inflammatory responses in human macrophages and its gene targets are more enriched for IBD GWAS hits than other pathways. Importantly, Stankey and colleagues presented data to indicate that this pathway can be targeted by MEK1 and MEK2 inhibitors in vitro and ex vivo in biopsies from people with active, untreated IBD. Although long-term MEK inhibition can lead to serious side-effects due to its role in multiple tissues, these data highlight how functional genomics can provide new insights into the pathophysiology of IBD and identify new drug targets.

The development and natural history of IBD in each affected individual is influenced by complex gene–environment interactions. In the November, 2024, issue of *eBioMedicine*, research by Chen and colleagues showed how air pollution is linked to increased susceptibility to ulcerative colitis, but not Crohn's disease. Among 450 000 participants in the UK Biobank, increased exposure to nitrogen oxides and particulate matter less than 2.5 μm in diameter was associated with risk of incident ulcerative colitis, especially in groups with increased exposure to air pollution and increased genetic risk or unhealthy lifestyle (ie, ever smoked, unhealthy diet, heavy alcohol consumption, or BMI ≥25 kg/m^2^). Further Mendelian randomisation and genome-wide methylation analyses provided evidence that epigenetic alterations in inflammatory loci might mediate this relationship.

Also, on the question of environmental effects, a report in the April, 2024, issue of *eClinicalMedicine* described a Danish nationwide population-based cohort study of 1,438,487 individuals aged 2–18 years between 1995 and 2020. In this cohort, exposure to high biodiversity and green space in early life was associated with reduced risk of Crohn's disease in adolescence. Conversely, exposure to agricultural land was associated with increased risk, supporting the notion that agricultural pesticides, fungicides, and other agents can lead to intestinal inflammation. However, no relationship was noted with ulcerative colitis. These observations highlight how the two inflammatory bowel diseases are interrelated but distinct, and can generate hypotheses for future research. Moreover, both studies emphasise the urgent need for environmental health research and environmental justice initiatives, as does a review published in *Gut* that described an increase in the incidence of IBD in low-income and middle-income countries that parallels increased chemical use and pollution.

Because of the non-specific symptoms, people with IBD can wait for a substantial amount of time for a definitive diagnosis. Advances in artificial intelligence offer opportunities to develop relevant new tools. A study by Kang and colleagues, published in the current issue of *eBioMedicine*, analysed alterations in Paneth cell density in whole-slide images of ileal tissue samples. A two-stage deep-learning model showed performance similar to expert pathologists but was much faster, and found reduced Paneth cell density in participants with Crohn's disease compared with healthy control individuals. People with the lowest 25% Paneth cell density had significantly shorter recurrence-free interval, suggesting potential for development of a prognostic biomarker.

After an IBD diagnosis, the disease course and outcomes are uncertain. Conventional management is based on gradual escalation of drug treatment until symptoms and the tendency to relapse are controlled. The PROFILE trial, published in *The Lancet Gastroenterology & Hepatology* in May, 2024, indicated that early combined immunosuppression with infliximab and an immunomodulator had better outcomes at 1 year than accelerated step-up treatment in the majority of people newly diagnosed with Crohn's disease. This trial was designed to test the utility of a prognostic biomarker derived from T-cell transcriptional signatures to stratify participants as likely to need treatment escalation. No clinical utility was observed in this case; whether other biomarkers can guide therapy choice for people with Crohn's disease or ulcerative colitis remains unknown.

Because of the increase in prevalence of IBD, reported in 2020 in *The Lancet Gastroenterology & Hepatology*, open questions need to be tackled to ensure effective treatment and satisfactory quality of life. At *eBioMedicine*, we offer a platform for high-quality research on all relevant aspects, from mechanisms through which genetic and environmental influences increase susceptibility to investigations of the pathophysiology, diagnostic and prognostic biomarkers, and early interventional studies.

